# Demand for digital skills, skill gaps and graduate employability: Evidence from employers in Malaysia

**DOI:** 10.12688/f1000research.148514.1

**Published:** 2024-04-25

**Authors:** Poh Kiong Tee, Ling Chai Wong, Morakinyo Dada, Bee Lian Song, Chee Pung Ng

**Affiliations:** 1 School of Marketing and Management, Asia Pacific University of Technology & Innovation, Kuala Lumpur, 57000, Federal Territory of Kuala Lumpur, Malaysia; 2 Faculty of Business and Communication, INTI International University, Nilai, 71800, Negeri Sembilan, Malaysia

**Keywords:** Digital transformation, Digital society, Digital economy, Digital skills, IR 4.0, Digital skill gaps, Graduate employability.

## Abstract

**Background:**

A major workforce inadequacy and the change in skill demanded have been observed due to the digital transformation. This study aims to identify the digital skills demanded by employers, focusing on exploring the skills gaps among the graduates that impact graduate employability.

**Methods:**

A cross sectional online survey was conducted among the companies registered with the Malaysian Productivity Corporation (MPC). Demand for digital skills was assessed using descriptive analysis of mean scores of the employers’ rating in digital skills at present and in future. A pair sample t-test was performed to explore the existence of skill gaps, by comparing the demand versus competencies of the graduates’ digital skills in the workplace.

**Results:**

Of the 393 responses collected, 376 responses were used for final analysis. The findings show that the current top three digital skills in demand are “information and data literacy”, “problem-solving”, and “digital content creation”. Whereas the top digital skills for future demand are “problem-solving”, “safety”, and “communication and collaboration”. In addition, the most significant (digital) skill gap is found in “communication and collaboration skills” followed by “problem-solving” and “safety” skills.

**Conclusions:**

These findings provide insights into the digital skills demanded by employers in IR 4.0, allowing the practitioners, education service providers and policymakers to do better planning on human capital management and training development. By focusing on identifying the current and future demand for digital skills while exploring the digital skill gaps among the graduates, this study provides insight into the employability skills required by the graduate before entering the job market.

## Introduction

The world has entered the new millennium of the Fourth Industrial Revolution (IR 4.0). New technologies and digitalisation significantly change nearly every aspect of our socioeconomic activities ( Khan *et al.,* 2021; Tee *et al.,* 2021). Traditional businesses are adopting new technologies to navigate a more extensive and challenging transformation of their operating processes; in the meantime, governments have made efforts to support the adoption of IR 4.0. The digital transformation affects the company’s operating structure and competitiveness and the need for human capital ( Rahmat *et al.,* 2021; Tee *et al.*, 2022). As the traditional model embraces new technologies and transforms into an intelligent environment, jobs will become more complex ( Caruso, 2018; Tsiligiris and Bowyer, 2021) while a significant workforce deficiency and shift in skill demand have been observed in the workplace [ Economic Planning Unit (EPU), 2021; Rahmat *et al.,* 2022]. Automation and digitalisation in the workplace are reshaping the employment landscape and bringing new challenges to human resources management, particularly in aligning the employees’ digital skills and competencies with the technological revolution [ Lee, 2019; World Economic Forum (WEF) 2016 and 2020]. Indeed, changes in the job requirements due to digital transformation and automation are the primary causes of job loss ( Kimball, 2021).

### Change in skill demand

In the transition to IR 4.0, the employees’ roles changed because of the changes to the work’s content, processes, and environment ( Lim *et al.,* 2021), which caused a shift in the skills demanded for employment. Beyond technical skills, digital skills in information management, communication, content creation, safety and problem-solving in the digital environment are in high demand ( Vuorikari *et al.,* 2016). As highlighted by Mercer (2019), recruiting or equipping employees with those digital skills is vital for supporting innovation and technology adoption for economic progress. Specifically, today’s work, employability, education, and social activities are being transformed by digitalisation.

Considering the skills demand arising from the dynamic transformations in the nature of work, employees, educational institutions, and employers encounter challenges in accurately predicting the requisite skill sets for employment and adequately preparing for the demands of the job market. Nevertheless, past studies [ Balcar *et al.,* 2018; Institute of Student Employers (ISE), 2018; Vuorikari *et al.,* 2016] revealed that graduates today lack the fundamental “digital skills” needed in the workplace. The gaps in digital skills among graduates pose a growing concern to educators, industry and the government, particularly the emergence of IR 4.0, which continues to disrupt business operations and occupations at a pace that outpaces employees’ ability to adapt. This circumstance may lead to unemployment or underemployment among graduates globally ( Deloitte, 2015). As highlighted by the World Economic Forum (2020), these digital skill gaps are expected to widen as employers increasingly prioritize digital competencies across various professions in the coming five years (2025).

### Problem statement and research objectives

Reviewing the statement above, it is evident that adopting IR 4.0 has substantially impacted the industry concerning the demand for skills, skills shortages, and unemployment. It is apparent that the future workforce needs to be technologically savvy; fresh graduates need to have a solid understanding of digital tools and technologies and job knowledge to add value to their work ( Khuraisah *et al.,* 2020). In a similar vein, the digital economy has been recognized as a key economic growth area (KEGA) in accomplishing Wawasan Kemakmuran Bersama 2030 (WKB 2030) and the Twelfth Malaysia Plan (RMKe-12), to transform Malaysia into a nation that achieves sustainable development with inclusive growth. The Malaysian government has implemented various measures to cultivate prospective digital talents and upskill or reskill the existing workforce to enhance the nation’s readiness to embrace the digital economy ( Economic Planning Unit, 2021).

Given the prevailing employment landscape, characterised by a demand for multiple skills ( Tee and Chan, 2016), one may contemplate whether graduates possess a comprehensive range of requisite abilities, specifically digital proficiencies aligned with the nation’s transition towards a digital economy. Indeed, it is always preferable for young graduates to acquire multiple skill sets, including technical and non-technical digital skills, to enhance their employment prospects ( Nair *et al.,* 2019; Rahmat *et al.,* 2022). Nevertheless, information on the actual skill mix and employers’ perspectives on the employability of graduates in IR 4.0 still needs to be explored. This study aims to fill the gaps by (1) discovering the digital skills demanded by employers currently and in the future to justify the change of skills demanded in the workplace due to IR4.0. Thereafter, (2) comparing the entry-level graduates’ level of competencies in digital skills against the important of digital skills to identify the existence of digital skill gaps among entry-level graduates that impact their employability. Most importantly, the views and experiences of employers hiring entry-level graduates will be gathered.

## Literature review

This study aimed to explore the perspective of employers concerning the demand for entry-level graduates’ digital skills. This study was grounded by the Resource-based view ( Barney, 1991) in relation to the recruitment of future graduates and sustaining their employability.

### Graduate employability

Unemployment and underemployment are pervasive challenges all nations face in light of the ongoing digital transformation [ International Labour Organisation (ILO), 2020]. Similarly, the issue of graduate unemployment in Malaysia has garnered attention in recent years, attributed to the large number of graduates being produced by various higher education institutions in Malaysia, including public and private colleges and universities, polytechnics, and vocational colleges ( Silva *et al.*, 2019; Tee *et al.,* 2022). The graduate unemployment rate in Malaysia was reported to increase to 4.1%, equivalent to 197,400 individuals in the same year (Department of Statistics Malaysia, DoSM, 2020).

Graduate employability pertains to individuals who have completed higher education and navigated their post-graduation life and employment ( Bennett, 2018; Tee *et al.,* 2019). Universities have been portrayed in this context as producing graduates with the skills employers need. However, previous studies ( Bennett, 2018; Clarke, 2018; Tsiligiris and Bowyer, 2021) found that, in addition to graduate skills and attributes, graduate employability depends on many other factors that are external to the educational process, such as changes in the skill demand as a result of the digital transformation. Also, most studies on employability skills were done before the dawn of the digital age. In contrast, the technical and non-technical skills that employers sought after would have changed over time due to the advancement of knowledge, technology, and globalisation. Therefore, it is imperative to investigate employers’ current and future skills demands and evaluate the present workforce’s (digital) skills and competencies to determine the presence of skills gaps or deficiencies. The findings of this study provide insight into the digital skills needed for graduates to succeed in their current and future jobs while lowering the youth unemployment rate.

### Demand for digital skills

Digital skills refer to the competencies required to effectively use information and communication technologies (ICT) in various aspects of life ( Gałan, 2022). These skills are essential in the 21st century and enable individuals to navigate and utilize digital tools and technologies for information management, communication, collaboration, critical thinking, creativity, and problem-solving ( Saputra *et al.,* 2021). Karacay (2017) found that employees must have information and communications technology (ICT) skills that facilitate collaboration and communication in the digital environment to perform tasks effectively in an I.R. 4.0-based system. Similarly, Van Laar (2020) identified the digital skills in the 21st century, inclusive of technical, informational, communicative, and collaborative competencies in a digital environment. Lee (2019) discovered that the graduates’ ability to use digital media to create creative content, information processing, and social networks is essential and frequently questioned during the job interview. However, Capone (2019) found that employers expect the graduates to have basic knowledge and understanding of operating digital technology to complete routine tasks instead of extensive statistical and programming knowledge. Van Laar (2020) further emphasises the importance of these digital skills as the prerequisites for individuals to thrive in today’s workforce.

The literature has consistently emphasized the critical need for digital skills across various domains in IR 4.0. Indeed, graduates must be equipped with these skills to effectively navigate the challenges and opportunities in the workplace presented in the digital era ( Bastian *et al.,* 2021; Joseph and Khan, 2020). Underpinned by the European Commission’s DigComp 2.0 framework ( Vuorikari *et al.,* 2016), this study conceptualizes the digital skills in the IR4.0 landscape consist of five categories: information and data literacy, communication and collaboration, digital content creation, safety, and problem-solving ( Table 1). Specifically, this study aims to identify and rank the set of digital skills currently demanded by employers and likely to be in demand in future (next five years) to answer the following research question:

**Table 1.** Digital competence framework.

Competence areas	Skills
1. Information and data literacy	1.1 Browsing, searching and filtering data, information and digital content. 1.2 Evaluating data, information and digital content. 1.3 Managing data, information and digital content
2. Communication and collaboration	2.1 Interacting through digital technologies. 2.2 Sharing through digital technologies. 2.3 Engaging in citizenship through digital technologies. 2.4 Collaborating through digital technologies.
3. Digital content creation	3.1 Developing digital content. 3.2 Integrating and re-elaborating digital content. 3.3 Copyright and licences. 3.4 Programming.
4. Safety	4.1 Protecting devices. 4.2 Protecting personal data and privacy. 4.3 Protecting health and well-being. 4.4 Protecting the environment
5. Problem solving	5.1 Solving technical problems. 5.2 Identifying needs and technological responses. 5.3 Creatively using digital technologies. 5.4 Identifying Digital Competence Gaps.

Source: Vuorikari *et al.* (2016).

*RQ1: “Which areas of digital skills demanded by the Malaysian employers currently and in the future?”*

### Digital skill gaps

The IR 4.0 has brought about significant changes in the job market, requiring individuals to possess a wide range of skills, making it challenging for fresh graduates to keep up and compete with more experienced candidates ( Rahmat *et al.,* 2021). Past studies have identified skill gaps in graduate employability within the framework of IR 4.0 and recognized that non-technical digital skills are regarded as significant qualities for enhancing employability in conjunction with technical skills ( Karacay, 2017; Khuraisah *et al.,* 2020). Indeed, cultivating digital skills is considered essential in higher education, given that they hold equal significance to technical skills ( Aidah *et al.,* 2019; Rahmat *et al.,* 2022; Tee *et al.,* 2019). Moreover, a disparity exists between the academic setting and the industry’s demands concerning graduate employability, underscoring the necessity to explore the skill gaps to tackle this concern ( Khuraisah *et al.,* 2020; Unni, 2016).

The Australian Institute of Management ( AIM, 2009) defines skill gap as the skill disparity between the skills required by the employers and the employees’ competencies in performing tasks. It reflects the employees need for more skills to meet job requirements ( Hogarth and Wilson, 2003; Rathelot and Van Rens, 2017) since the presence of skill gaps is the main contributing factor towards unemployment, thereby having significant financial implications for a nation’s economic development ( Rathelot and Van Rens, 2017; Suleman and Laranjeiro, 2018; WEF, 2016). Employers’ viewpoints should be taken into account in order to address the skills gaps ( Balcar *et al.,* 2018; Tomlinson, 2017; Truong *et al.,* 2018), and most of the past studies employed the same approach to assess the magnitude of the skills gap by comparing employers’ rating score of the important of different skills (i.e., soft skills, technical skills, and digital skills) with the level of satisfaction on the employees’ performance ( Abbasi *et al.,* 2018). Through the skill gaps analysis, companies can assess the candidate’s competencies during the recruitment process by matching the skills possessed by candidates with the tasks required, thereby reflecting the employability skills of the individuals.

This study employs a methodology similar to the previous researchers ( Abbasi *et al.,* 2018; Kenayathulla *et al.,* 2019), wherein employers’ expectations of the skills in demand were compared with the employee’s level of competence in similar skills. This study’s findings can ascertain potential gaps between the skills demanded and the skills possessed by the graduates in justifying their employability. Accordingly, this study aims to answer the following questions:

*RQ2: “What are the level of digital competencies of entry-level graduate employees in Malaysia?”*

*RQ3: “What are the areas of digital skills gap among the entry-level graduate employees in Malaysia?”*

## Methods

This study examines the employers’ demand for digital skills against the skill competencies of the entry-level graduate workforce with less than two years of working experience. Areas of digital skill gaps were identified by comparing the employers’ perceptions of the essential digital skills against the digital competencies of the employees (i.e., graduates) in performing their tasks.

### Ethics and consent

This study was approved by the Ethics Committee of Asia Pacific University of Technology and Innovation, Kuala Lumpur, Malaysia (APUFE/02/2023) in July 12, 2023. All of the informants gave written and oral informed consent to participate in the study. Under the Personal Data Protection Act (PDPA) 2010, the researchers assure that all information collected is kept confidential and merely for the academic purposes.

### Population and sample

The population in this study is the companies registered with the Malaysian Productivity Corporation (MPC) office. MPC is a statutory body govern by the Malaysian Ministry of Investment, Trade and Industry (MITI), aims to drive national productivity at all levels. The list of companies registered with MPC was obtained from MPC. The companies in Klang Valley (i.e., Selangor and Kuala Lumpur), Johor and Penang were selected for this study since these states had the most firms located and registered with MPC. Eight hundred ninety-five online questionnaires were distributed, and 393 responses were collected using a simple random sampling technique (i.e., 44% response rate). After the data screening process, 376 responses, including 215 from Klang Valley, 58 from Johor and 103 from Penang, were used for further analysis. In addition, the sectors in which companies operate are mainly the service sector (43%), manufacturing (32%), retail (18%) and other sectors (7%).

### Measures

This study aims to identify the digital skills demanded by employers at present and in the future while investigating the presence of the digital skills gaps among Malaysian entry-level graduate employees by comparing the employers’ expectations against the skill competencies of the graduate employees in performing their work. The measuring items of the digital skills were adapted from the European Commission’s Digital Competence 2.0 ( Vuorikari *et al.,* 2016). Nineteen (19) items were adapted to measure the five (5) categories of digital skills (i.e., information and data literacy, communication and collaboration, digital content creation, safety, and problem-solving). One (1) question asks the employers to indicate the importance of the three common employability skills (i.e., technical skills, soft skills and digital skills). Another (1) question to measure graduate employability is asking employers to indicate the importance of digital skills when hiring fresh graduates. All items were measured using a 5-point Likert scale: “1= not at all important to 5= very important”.

### Data analysis

A descriptive analysis was conducted using IBM SPSS v26 ( https://www.ibm.com/spss), to interpret the respondents’ (i.e., the employers) demographic profile and the phenomenon under study. The data was assessed based on the mean scores of the employers’ rating on the importance of digital skills required for the graduate employees to perform their tasks at present and in the future. The digital skill category with the highest mean score ranked one (1), was deemed the most important.

A compare mean analysis using IBM SPSS v26, was conducted to identify the differences in mean scores between skills demanded at present with the level of competence to signify the magnitudes of said skill gaps. A higher mean gap value signifies a significant digital skill gap, and a negative value indicates that graduate employees lack the competencies to meet the employer’s expectations and vice versa. Lastly, a paired sample t-test was conducted to justify the existence of digital skills gaps among the entry-level graduate employees.

## Results

The data collected was analysed using descriptive analysis (i.e., means and standard deviation) and inferential statistics via comparison of means using a t-test. In addressing the level of preference for technical skills, soft skills and digital skills among employers, digital skills were regarded as very important and extremely important by 99.5% of the respondents (n=374), followed by soft skills (95.5%, n=359) and technical skills (78.5%, n=295). This result indicates that digital skills are highly demanded by employers in today's workplace, particularly by entry-level graduates.

### Digital skills in demand at present and in the future

The respondents (i.e., employers) were asked to rate the digital skills demanded at present and in the future using a 5-point Likert scale - “1= not at all important to 5= extremely important”. The results in Table 2 summarise the current and future demand for the five categories of digital skills. All these digital skills have mean scores greater than 3.60 (out of 5), denoting that the employers viewed all these skills as “fairly important” and nearing “very important” in the current state. “Information and data literacy” (μ= 4.048), “digital content creation” (μ=4.015) and “problem-solving skills” (μ=3.999) are the three most demanded digital skills by employers at present. Specifically, the mean score for the measuring item “integrating and re-elaborating digital content” has the highest mean (μ=4.213) and the item “engaging in citizenship through digital technologies” has the lowest mean score (μ=3.626).

**Table 2.** Digital Skills in Demand (Current and Future).

Categories/Items	Current		Future	
	Mean	Rank *	Mean	Rank *
**Information and data literacy**	**4.048**	*1*	**4.123**	*5*
1. Browsing, searching and filtering information and digital content.	3.858		4.045	
2. Evaluating data, information and digital content.	4.133		4.177	
3. Managing data, information and digital content.	4.075		4.146	
**Communication and Collaboration**	**3.640**	*5*	**4.431**	*3*
1. Interacting through digital technologies.	3.662		4.500	
2. Sharing through digital technologies.	3.631		4.449	
3. Engaging in citizenship through digital technologies.	3.626		4.343	
4. Collaborating through digital technologies.	3.635		4.423	
**Digital content creation**	**4.015**	*2*	**4.407**	*4*
1. Developing digital content.	3.981		4.399	
2. Integrating and re-elaborating digital content.	4.213		4.414	
3. Copyright and licenses.	3.866		4.354	
4. Programming.	4.038		4.413	
**Safety**	**3.771**	*4*	**4.518**	*2*
1. Protecting devices.	3.737		4.541	
2. Protecting personal data and privacy.	3.803		4.515	
3. Protecting health and well-being.	3.813		4.467	
4. Protecting the environment.	3.732		4.334	
**Problem-solving**	**3.999**	*3*	**4.553**	*1*
1. Solving technical problems.	4.000		4.555	
2. Identifying needs and technological responses.	3.981		4.552	
3. Creatively using digital technologies.	3.983		4.549	
4. Identifying digital competence gaps.	4.031		4.566	

* Note: “1= very important to 5= very unimportant”.

On the other hand, all the digital skills are viewed as “very important” and nearing “extremely important” in the future (mean score above 4.0). The three areas of digital skills that are highly demanded in the future are “problem-solving skills” (μ=4.553), “safety” (μ=4.518), and “communication and collaboration” (μ=4.431) ( Table 2). Notably, the results show the difference in the demand for digital skills at present and in the future. Both “safety” (μ=3.771) and “communication and collaboration” (μ=3.640), perceived as “fairly important” at present, were rated nearing “extremely important” in the future. It is observed that the demand for “information and data literacy” and “digital content creation” have been normalised, moving them from first and second in current demand to fourth and fifth in future demand.

Further study on the rising demand for digital skills in the future, the three categories of digital skills that show a significant increase in mean scores of above 0.50 when comparing the current and future demand are: “communication and collaboration”, “safety” and “problem-solving skills” (change in mean scores: 0.791, 0.747 and 0.554 respectively). Specifically, the mean scores for the measuring item “identifying digital competence gaps” has the highest mean (μ=4.566) and the item “browsing, searching and filtering information and digital content” has the lowest mean score (μ=4.045). The results imply that the digital skills demanded have changed over time due to the advancement of knowledge and technology in IR 4.0.

### Digital competencies and digital skill gaps

The entry-level graduate employee’s level of competencies is rated on a 5-point scale – “1=very poor to 5=very good”. The results shown in Table 3 revealed that the existing graduate employees scored “average” to nearing “poor” competence in most of the digital skills (mean score of 2.681 to 3.411, respectively). These employees are poor in “communication and collaboration" skills (μ=2.713), “safety” skills (μ=3.000), and “problem-solving” (μ=3.135).

**Table 3.** Level of competence in digital skills.

Categories/Items	Competence	
	Mean	Rank *
**Information and data literacy**	**3.391**	*5*
1. Browsing, searching and filtering information and digital content.	3.411	
2. Evaluating data, information and digital content.	3.403	
3. Managing data, information and digital content.	3.374	
**Communication and Collaboration**	**2.713**	*1*
1. Interacting through digital technologies.	2.701	
2. Sharing through digital technologies.	2.681	
3. Engaging in citizenship through digital technologies.	2.713	
4. Collaborating through digital technologies.	2.724	
**Digital content creation**	**3.275**	*4*
1. Developing digital content.	3.302	
2. Integrating and re-elaborating digital content.	3.307	
3. Copyright and licenses.	3.232	
4. Programming.	3.289	
**Safety**	**3.000**	*2*
1. Protecting devices.	2.970	
2. Protecting personal data and privacy.	3.061	
3. Protecting health and well-being.	2.975	
4. Protecting the environment.	2.995	
**Problem-solving**	**3.135**	*3*
1. Solving technical problems.	3.041	
2. Identifying needs and technological responses.	3.233	
3. Creatively using digital technologies.	2.987	
4. Identifying digital competence gaps.	3.089	

* Note: “1= lowest to 5= highest”

A paired sample t-test was conducted to identify digital skills gaps by comparing the differences in mean scores between the skills demanded (level of importance) against the level of competencies. The difference in mean score (i.e., skill gaps) indicates if graduates possess digital skills that meet employers’ expectations. A negative value indicates that the employees lack the competencies to meet the employer's expectations and vice versa.

As shown in Table 4, the p-value (2-tailed) is less than 0.05, concluding that there is a significant difference in the skill demand scores and competencies scores for pairs 1-5 (i.e., the five categories of digital skills). All negative values indicate the presence of skill gaps among the existing entry-level graduates in all identified digital skills in this study. Specifically, the highest gap exists in “communication and collaboration” (μ=-0.927), followed by “problem-solving” (μ=-0.864), “safety” (μ=-0.771), “digital content creation” and “information and data literacy” (μ=- 0.740 and μ=-0.654 respectively).

**Table 4.** Results for paired sample t-test and digital skill gaps.

	Mean	SD	95% CI		t-value	Sig
			Lower	Upper		
Information and data literacy	-0.654	0.574	-0.715	-0.598	-19.165	<0.01
Communication & collaboration	-0.927	0.785	-0.960	-0.711	-28.415	<0.01
Digital content creation	-0.740	0.632	-0.815	-0.638	-23.814	<0.01
Safety	-0.771	0.707	-0.884	-0.698	-23.986	<0.01
Problem-solving	-0.864	0.755	-0.916	-0.719	-25.429	<0.01

## Discussion

The results in this study show that the majority of the employers (99.5%) preferred their entry-level graduate employees to possess digital skills compared to technical skills (78.5%) and soft skills (95.5%). Similarly, most employers (μ=4.5 out of 5) consider digital skills “extremely important” in hiring entry-level graduates. These findings are consistent with past studies that emphasise the high demand for digital skills for graduate employability in Malaysia ( Khan *et al.,* 2021; Khuraisah *et al.,* 2020; Rahmat *et al.,* 2022). Although hard skills (i.e., technical skills) are in demand during recruitment, digital skills are highly valued by employers, especially in coping with the technological advancement in IR 4.0 ( Chen *et al.,* 2018; Jewell *et al.,* 2020; Malik and Venkatraman, 2017; Siddoo *et al.,* 2019).

### Demand for digital skills at present

The results indicate that, at present, these digital skills are fairly important and (or) important to the employers. Among the five categories of digital skills, “information and data literacy”, “digital content creation”, and “problem-solving skills” are in high demand by employers at present. Considering the increasing adoption of new technology and information communication technology (ICT) in the workplace, the demand for information and digital literacy, as well as technology knowledge, is high at present ( Khuraisah *et al.,* 2020; Valdés *et al.,* 2018). Thus, entry-level graduates are expected to be capable of “analysing and interpreting data and information” as well as “organising and retrieving data and information” in the digital environment, whereas “browsing and searching information” is considered a less important digital skill. This finding is supported by past studies ( Jewell *et al.,* 2020; Saunders, 2018), which found that browsing, searching and locating data are considered lower-order skills generally performed by clerical staff instead of graduate employees.

Besides, digital content creation is also in high demand at present due to the rise of new information visualisation tools and the rapid growth of social media ( Vuorikari *et al.,* 2016). Consistently, past studies also highlighted the importance of digital content creation for knowledge workers to support their everyday digital practices in presenting, sharing, and reusing information. ( Brown *et al.,* 2016; Venkitachalam and Bosua, 2019). Another vital skill highly demanded in this digitisation era is problem-solving skills. While employers do not expect fresh graduates to possess broad expertise upon entering the job market, they expect the employees to be able to solve technical problems when operating devices in digital environments ( Spector and Kinshuk, 2011). Jewell *et al*. (2020) and Aidah *et al.* (2019) suggest that graduates who can think critically and creatively while using digital tools to solve problems within digital contexts will create a competitive advantage over others.

### Demand for digital skills in the future

As technology evolves, the emergence of new technologies introduces additional layers of complexity along with ongoing changes to the demand for digital skills in the future. The findings show a significant increase in the demand for digital skills in the future, in which all categories of digital skills are viewed as nearing “extremely important” (μ=4.5 out of 5) in the future. Consistently, the digital skills demanded by employers have changed along with the emergence of new technologies. “Problem-solving skills”, “safety”, and “communication and collaboration” are rated as the top three digital skills demanded in the future. The demand for “information and data literacy” and “digital content creation” have been normalised. These findings are consistent with past studies ( Jewell *et al.,* 2020; Khuraisah *et al.,* 2020; Suarta and Suwintana, 2021), which addressed the importance of digital problem-solving skills as an instrumental in harnessing the potential of digital innovations while mitigating their risks. Also, the constant emergence of new issues associated with new information and communication technologies elevates the significance of problem-solving as a differentiator in the workforce.

Along with problem-solving skills, competence in protecting the company’s devices, personal data and privacy (i.e., “safety”) was perceived as an extremely important digital skill in the future. According to Rahmat *et al.* (2022), companies in digital transformation seem to utilise many digital tools and platforms to integrate internal systems with customer interfaces in their business operation. These companies are exposed to higher risks in data protection and violation of personal privacy. Hence, most employers demand future graduates to have basic “safety” skills to create a safe digital environment in the workplace ( Frania, 2014; Tomczyk, 2019). Another vital skill highly demanded in the future is “communications and collaborations”. The Employers expect future graduates to have functional digital literacy and the ability to use core computer applications in data processing and information sharing through digital technologies. Entry-level graduates in this era must and should be able to communicate effectively in all digital forms ( Chen *et al.,* 2018; Othman *et al.,* 2018). Indeed, virtual communication and collaboration are two vital skills needed in the future workforce ( Ellahi *et al.,* 2019; Picatoste *et al.,* 2018; Siddoo *et al.,* 2019).

### Digital skill competencies and skills gaps of graduate employees

The results indicate that the existing graduate workforce needs more competence in digital skills to meet their employers’ expectations. Gaps were found in all categories of digital skills. Notably, the finding shows that “information and data literacy” (i.e., digital literacy) was ranked relatively low in the skills gap compared to other digital skills. This statement is due to the fact that new generations are inherently tech-savvy, and these younger generations (graduates) tend to quickly adapt to new digital tools and platforms ( Helsper and Eynon, 2010; Kennedy *et al.,* 2008). The entry-level graduates’ ability to use digital tools (i.e., browsing and searching information) is increasingly seen as a foundational skill, similar to reading and writing, resulting in a small gap in “information and data literacy” compared to other digital skills.

On the other hand, the highest digital skill gaps were found in “communication and collaboration” and “problem-solving”. Notably, both are highly demanded skills in future employment. This finding has some consonance with the skills gaps identified in past studies. According to Nardo *et al.* (2020) and Rahmat *et al.* (2021), employees are expected to have (non-technical) knowledge and skills to use digital tools and technologies, interact with modern interfaces, and be aware of digital security in the IR 4.0 environment, to complement the lack of technical skills. However, most employers are unsatisfied with existing graduates’ non-technical skills, including communication and problem-solving skills, where the graduates need to adapt to the real working environment ( Suleman and Laranjeiro, 2018). In a similar vein, a study on Malaysian graduate work-readiness by Verma *et al.* (2018) discovered that the university curriculum at present is unable to foster the skills needed in the workplace, which has a notable impact on students’ capacity to communicate effectively and a lack in critical thinking and problem-solving skills.

### Implications of the study

This study obtained feedback from employers on areas of digital skills that graduates need to enhance their employability in the new age. It is of practical relevance as the world is moving into the new age of IR 4.0 while there are changes in technology and skills demand. Following the government's aspiration to transform Malaysia into a digitally enabled and technology-driven nation in a digital economy, the findings of this study help various stakeholders (i.e., higher education providers and the authorities) develop a successful plan by highlighting the digital skills required for better employment opportunities. Employers and human resource professionals can use this information to inform their recruitment and talent development plans, specifically to create training and development program to close the identified skill gaps. A holistic approach to skill development beyond technical proficiency is also required, given the future emphasis on problem-solving, safety, and communication skills in a digital context.

Moreover, the shift in the demand for digital skills that has been observed offers direction for curricular development in higher education, including modules that emphasize problem-solving, safety, and communication to equip graduates better to meet industry standards. Collaboration between academic institutions and industry can also help align curriculum with practical business demands. Lastly, the empirical findings in this study can also be used as a reference for the policymakers in Ministry of Education (MOE), Ministry of Human Resources (MOHR), Human Resources Development Foundation (HRDF) and training providers to develop policies and strategies for study/training curricula and methodology to ensure the related skills are imparted.

Theoretically, this study extended from the employers’ perspective, as the “demand” in selecting candidates based on the required skills concerning recruiting future graduates and sustaining their employability. Underpinned by the Resource-based view, the findings of this study elucidate the graduate employment landscape by identifying the digital skills graduates must possess and whether these digital skills can be considered as a firm's resource, which may influence employers' hiring decisions in the 4IR environment.

### Limitations and direction for future studies

This study has limitations that should be considered while interpreting the findings, notwithstanding the contributions and implications. The study focused on specific regions (states) within Malaysia, hence the results might not be easily applicable to other nations with diverse technology and economic environments. A more thorough understanding of the demand for digital skills can result from replicating the study in different contexts.

The data gathered was based on self-reported assessments of the employers. Due to social desirability or incorrect question interpretation, bias could be present. Using additional techniques, such as conducting interviews or carrying out direct observation, could provide a more comprehensive understanding of the skills and competences. Instead of using an objective evaluation, the study’s method for measuring skills depended on self-evaluation. To verify the self-reported data, future studies can include performance reviews or assessments from independent parties. Future research could examine the demand for digital skills within different sectors to provide tailored insights, given the distinct demands across various sectors. Moreover, this study offers an overview of abilities and skill demand at a specific period. A longitudinal study could monitor shifts in competence and demand over time, giving researchers a more dynamic knowledge of digital skill trends.

This study was primarily based on the premise that enhancing digital skills able to increase the entry-level graduates’ employability. However, personal qualities of the graduates seem to play crucial roles in their employment ability because higher quality person (i.e., a person with better soft skills) can contribute more to his or her employment ability. Moreover, recent studies have shown personal qualities as an important concern during employment ( Al Asefer and Zainal Abidin, 2021; Tsiligiris and Bowyer, 2021). This study focused on the demand for digital skills, digital skills gaps and employability without contemplating the roles of personal qualities. We see this as a significant limitation of this study and suggest future studies to explore this aspect.

## Conclusion

In light of the growing significance of digital skills in today’s society, this study offers an essential viewpoint concerning the demand for digital skills and the digital skill gaps in IR 4.0. The model developed in this study aims to specify metrics for evaluating digital skills that improve employability in Malaysia. Beyond the current study, future research will examine additional metrics for digital skills that affect graduate employability in Malaysia.

### Ethics and consent

This study was approved by the Ethics Committee of Asia Pacific University of Technology and Innovation, Kuala Lumpur, Malaysia (APUFE/02/2023) in July 13, 2023. All of the informants gave written and oral informed consent to participate in the study. Under the Personal Data Protection Act (PDPA) 2010, the researchers assure that all information collected is kept confidential and merely for the academic purposes.

## Data Availability

Figshare: Digital Skills_ Questionnaire & Dataset. figshare. Dataset. https://doi.org/10.6084/m9.figshare.25540798.v2 ( Tee, 2024). This project contains the following underlying data:
• Data set: Full Data TPK_DS_092023.xlxs. • Letter for Participation & Questionnaire_TPK_DS.pdf. Data are available under the terms of the Creative Commons Zero “No rights reserved” Attribution 4.0 International (CC BY 4.0)
