# Supplementary material for: Demand for digital skills, skill gaps and graduate employability: Evidence from employers in Malaysia
Source: F1000Res. 2024 Apr 25;13:389. [Version 1] doi: 10.12688/f1000research.148514.1 (PMC11387931; doi:10.12688/f1000research.148514.1)
Supplement: Peer review history

# Reviewer report: Reviewer response for version 1

**DOI:** 10.5256/f1000research.162834.r314046

**Published:** 2024-09-20

**Authors:** Samia Mokhtar Shahpo

**Affiliations:** 1 Imam Abdulrahman Bin Faisal University, Dammam, Eastern Province, Saudi Arabia

The subject of skills necessary to employ graduates is one of the topics that receive attention from the relevant authorities. The researchers formulated a title expressing the content of the research and provided a complete and interesting abstract. The research problem is specific and clear, and the questions are compatible with the objectives of the study. The methodology used is appropriate, and the statistical methods used are appropriate for the study design. Reviewing and discussing the results were logical and supported by evidence. However, the terms in the study were not defined in a specific, measurable procedural form. Benefits from previous studies have not been presented in the present study. For results, I recommend adding open-ended questions to the questionnaire or using interviews with employers and not just using the closed questionnaire.

Is the work clearly and accurately presented and does it cite the current literature?

Yes

If applicable, is the statistical analysis and its interpretation appropriate?

Yes

Are all the source data underlying the results available to ensure full reproducibility?

Yes

Is the study design appropriate and is the work technically sound?

Yes

Are the conclusions drawn adequately supported by the results?

Yes

Are sufficient details of methods and analysis provided to allow replication by others?

Yes

Reviewer Expertise:

EducationLearnersPsychology

I confirm that I have read this submission and believe that I have an appropriate level of expertise to confirm that it is of an acceptable scientific standard.

## References

[1] : Demand for digital skills, skill gaps and graduate employability: Evidence from employers in Malaysia. *F1000Research* .2024;13: 10.12688/f1000research.148514.1 10.12688/f1000research.148514.1 PMC1138793139262836

# Reviewer report: Reviewer response for version 1

**DOI:** 10.5256/f1000research.162834.r303733

**Published:** 2024-09-18

**Authors:** Md Jahangir Alam

**Affiliations:** 1 University of Dhaka, Dhaka, Dhaka Division, Bangladesh

This research has a very high significance. However, I recommend the authors review the following articles:.

 As the conceptual and theoretical frameworks are missing, I recommend developing a conceptual framework for this study. To align the result with the discussion, there is still room for improvement. The auditors started with RQ2 and RQ3, but I did not find RQ1.  

1. Digitalization of higher education to achieve sustainability: Investigating students’ attitudes toward digitalization in Bangladesh
2. Skills development for graduate employability in Bangladesh: Japanese language in TVET program
3. e-Learning as a Doubled-Edge Sword for Academic Achievements of University Students in Developing Countries: Insights from Bangladesh
4. Importance of Skills Development for Ensuring Graduates Employability: The Case of Bangladesh
5. Skills development for sustainable career planning in Bangladesh: the case of gender equality in education
6. Future-readiness and employable ICT skills of university graduates in Bangladesh: an analysis during the post-covid era
7. Japanese investment for skills development program in attaining sustainable employability: The case of Bangladesh
8. Sustainable employment for vocational education and training graduates: the case of future skills matching in Bangladesh

Is the work clearly and accurately presented and does it cite the current literature?

Partly

If applicable, is the statistical analysis and its interpretation appropriate?

Partly

Are all the source data underlying the results available to ensure full reproducibility?

Yes

Is the study design appropriate and is the work technically sound?

Yes

Are the conclusions drawn adequately supported by the results?

Partly

Are sufficient details of methods and analysis provided to allow replication by others?

Partly

Reviewer Expertise:

The authors need to develop the conceptual framework of this research The result and findings do not align and I recommend reviewing further article.

I confirm that I have read this submission and believe that I have an appropriate level of expertise to confirm that it is of an acceptable scientific standard, however I have significant reservations, as outlined above.

# Reviewer report: Reviewer response for version 1

**DOI:** 10.5256/f1000research.162834.r288764

**Published:** 2024-09-10

**Authors:** Noor Aslinda Abu Seman

**Affiliations:** 1 Universiti Tun Hussein Onn Malaysia, Parit Raja, Malaysia

Overall, this study is well-executed and provides significant insights into the digital skills demanded by employers in Malaysia, along with highlighting the skill gaps among graduates. The methodology is robust, and the findings are presented clearly. However, there are areas where improvements can be made:

1. **Graduate Employability Section**
  - **Current Text:** "The graduate unemployment rate in Malaysia was reported to increase to 4.1%, equivalent to 197,400 individuals in the same year."
  - **Improvement:** Please specify the year to which this data refers. For example, "The graduate unemployment rate in Malaysia was reported to increase to 4.1%, equivalent to 197,400 individuals in 20xx." This adds clarity and context for the reader.
2. **Table Placement**
  - **Current Placement:** Table 1 is not optimally placed in the manuscript.
  - **Improvement:** Table 1 should be placed immediately after the Research Question 1 (RQ1) statement. This will help in better aligning the table with the relevant section of the text, making it easier for readers to follow and understand the presented data.

Is the work clearly and accurately presented and does it cite the current literature?

Yes

If applicable, is the statistical analysis and its interpretation appropriate?

Yes

Are all the source data underlying the results available to ensure full reproducibility?

No source data required

Is the study design appropriate and is the work technically sound?

Yes

Are the conclusions drawn adequately supported by the results?

Yes

Are sufficient details of methods and analysis provided to allow replication by others?

Yes

Reviewer Expertise:

Management, Marketing, Green Supply Chain Management, SMEs

I confirm that I have read this submission and believe that I have an appropriate level of expertise to confirm that it is of an acceptable scientific standard.

# Reviewer report: Reviewer response for version 1

**DOI:** 10.5256/f1000research.162834.r274442

**Published:** 2024-05-25

**Authors:** Melinde Coetzee

**Affiliations:** 1 Department Industrial & Organisational Psychology, University of South Africa, Pretoria, Gauteng, South Africa

The study is of value and high relevance to the field of employability, especially gathering views from employers hiring entry level graduates. The problem statement is well-justified. The study aims are clear.

 Contextualization of study: The authors focused primarily on 4IR as context. The recommendation is to critically review and elaborate briefly on the relevance of the study to 5IR technology as well. Apart from 4IR informing the change in skill demand, the authors should critically review the 5IR’s (the future) impact on skills demands

 Check for grammatical errors- e.g. sentence in paragraph before literature review

 Thereafter, (2) comparing the entry-level graduates’ level of competencies in digital skills against the

important of digital skills to…… - should read importance

 Literature review- the authors should briefly justify why and how the study is grounded by the Resource-based view 

 Although the paper reflects relatively recent sources from the research literature (2020-2022), one would like to see more recent research literature added (2023/2024) to strengthen the contemporary relevance of the work.

 Table 1 used a 2016 source of digital competence as framework- what are current digital competence frameworks for 5IR? Is the 2016 competence framework still of relevance? The authors need to justify their assumptions of digital competencies needed by integrating more recent research literature corroborating the 2016 framework and measure.

 Overall, the paper is well-written and arguments flow logically

 The research methodology is suitable for the research aims

 The discussion is enriching and convincing.

Is the work clearly and accurately presented and does it cite the current literature?

Partly

If applicable, is the statistical analysis and its interpretation appropriate?

Yes

Are all the source data underlying the results available to ensure full reproducibility?

No source data required

Is the study design appropriate and is the work technically sound?

Yes

Are the conclusions drawn adequately supported by the results?

Yes

Are sufficient details of methods and analysis provided to allow replication by others?

Yes

Reviewer Expertise:

NA

I confirm that I have read this submission and believe that I have an appropriate level of expertise to confirm that it is of an acceptable scientific standard.
